# Rare case of multiple brain abscess *– Scedosporium apiospermum*

**DOI:** 10.1186/1471-2334-12-S1-P59

**Published:** 2012-05-04

**Authors:** Subin Mathew, Ravi Mohan Rao, S Raghavendra, BA Chandramouli, Ravindra Kamble, Sreedharan Athmanathan

**Affiliations:** 1 Department of Neuroscience, Vikram Hospital, Bangalore, India; 2 Department of Radiology, Vikram Hospital, Bangalore, India; 3 Department of Microbiology, Vikram Hospital, Bangalore, India

## Introduction

*Scedosporium apiospermum* is the asexual form of fungus *Pseudallescheria boydii* which is commonly found in soil and sewage. It usually causes cutaneous infection (mycetoma) and otitis externa and in rare circumstances causes invasive disease in immunocompromised patients, which can involve the central nervous system with devastating prognosis. We report a case of brain abscess caused by *S. apiospermum*.

## Case presentation

A 9 year old boy with a history of hydrocephalus with multiple VP shunt surgeries, presented to us with history of unsteadiness of gait since ten days, headache and vomiting for a period of 5 days with an episode of fall. Initial MRI showed trapped fourth ventricle with dilated left occipital horn. Endoscopic fenestration of lateral ventricle cyst and microsurgical fenestration of trapped fourth ventricle was performed. A repeat MRI done on the fourth day of admission showed multiple small ring enhancing lesions scattered in right cerebral hemisphere. He was started on antibiotics. MRI done on the sixth day revealed increase in size of the lesions for which he underwent decompressive craniectomy, anterior temporal lobectomy and the specimen studded with multiple hemorrhagic lesion was sent for histopathology and microbiological evaluation. His condition worsened and he succumbed to the illness on the eight day after admission. Culture revealed *S. apiospermum*.

## Discussion

Intracranial fungal infections due to *S. apiospermum* are difficult to treat and are often fatal. Maintaining a high clinical suspicion, early diagnosis, appropriate antifungal therapy (e.g. voriconazole) and combined surgical interventions may improve prognosis.

